# Quantifying Anti-HIV Envelope-Specific Antibodies in Plasma from HIV Infected Individuals

**DOI:** 10.3390/v11060487

**Published:** 2019-05-28

**Authors:** Sanket Kant, Ningyu Zhang, Jean-Pierre Routy, Cécile Tremblay, Réjean Thomas, Jason Szabo, Pierre Côté, Benoit Trottier, Roger LeBlanc, Danielle Rouleau, Marianne Harris, Franck P. Dupuy, Nicole F. Bernard

**Affiliations:** 1 Research Institute of the McGill University Health Centre (RI-MUHC), Montreal, QC H3A 3J1, Canada; sanket.kant@mail.mcgill.ca (S.K.); crystal.zhang.1995@hotmail.com (N.Z.); jean-pierre.routy@mcgill.ca (J.-P.R.); dura007@hotmail.fr (F.P.D.); 2 Division of Experimental Medicine, McGill University, Montreal, QC H4A 3J1, Canada; 3 Infectious Diseases, Immunology and Global Health Program, Research Institute of the McGill University Health Centre, Montreal, QC H4A 3J1, Canada; 4 Division of Hematology, McGill University Health Centre, Montreal, QC H4A 3J1, Canada; 5 Chronic Viral Illness Service, McGill University Health Centre, Montreal, QC H4A 3J1, Canada; 6 Centre de Recherche du Centre Hospitalier de l’Université de Montréal (CRCHUM), Montreal, QC H2X 3H8, Canada; c.tremblay@umontreal.ca; 7 Department of Microbiology Infectiology and Immunology, University of Montreal, Montreal, QC H3C 3J7, Canada; 8 Clinique médicale l’Actuel, Montréal, QC H2L 4P9, Canada; rejean.thomas@lactuel.ca (R.T.); jason.szabo@lactuel.ca (J.S.); 9 Clinique Médicale Quartier Latin, Montréal, QC H2L 4E9, Canada; dufresne.cote@sympatico.ca (P.C.); bentrotte@gmail.com (B.T.); 10 Clinique Médicale Opus, Montréal, QC H3A 1T1, Canada; rogerpleblanc@me.com; 11 Département de microbiologie, infectiologie et immunologie, Faculté de médecine, Université de Montréal, Montréal, QC H2L 4M1, Canada; danielle.rouleau@umontreal.ca; 12 St. Paul’s Hospital, Vancouver, BC V6Z 1Y6, Canada; mharris@cfenet.ubc.ca

**Keywords:** HIV, HIV envelope, antibodies, flow cytometry, ELISA, CEM.NKr.CCR5

## Abstract

Quantifying HIV Envelope (Env)-specific antibodies in HIV^+^ plasma is useful for interpreting antibody dependent cellular cytotoxicity assay results. HIV Env, the only viral protein expressed on the surface of infected cells, has a native trimeric closed conformation on cells infected with wild-type HIV. However, CD4^+^ uninfected bystander cells in HIV^+^ cell cultures bind gp120 shed from HIV^+^ cells exposing CD4-induced epitopes normally hidden in native Env. We used flow-cytometry based assays to quantify antibodies in HIV^+^ plasma specific for native trimeric Env or gp120/CD4 conjugates using CEM.NKr.CCR5 (CEM) cells infected with HIV (iCEM) or coated with recombinant gp120 (cCEM), as a surrogate for gp120^+^ HIV^-^ bystander cells. Results from both assays were compared to those of a plate-based ELISA to monomeric gp120. The levels of Env-specific antibodies to cCEM and iCEM, measured by flow cytometry, and to gp120 by ELISA were positively correlated. More antibodies in HIV^+^ plasma recognized the gp120 conformation exposed on cCEM than on iCEM. Comparisons of plasma from untreated progressors, treated progressors, and elite controllers revealed that antibodies to Env epitopes were the lowest in treated progressors. Plasma from elite controllers and untreated progressors had similarly high levels of Env-specific antibodies, despite elite controllers having undetectable HIV viral loads, while untreated progressors maintained high viral loads.

## 1. Introduction

The RV144 or Thai HIV vaccine trial was the first to show a significant, though modest (31.2%), efficacy in protecting against HIV infection [1]. In this trial, broadly neutralizing antibodies (BnAbs) and cytotoxic T lymphocyte responses were not implicated in HIV protection. The presence of human immunoglobulin G (IgG) antibodies (Abs) specific for the V1/V2 loop of HIV Envelope (Env) was associated with protection, provided that human immunoglobulin A Abs with overlapping specificity were absent [2,3,4]. Secondary analyses of the results of the RV144 trial and the earlier VAX004 vaccine trial found an inverse correlation between Fc mediated effector functions, such as antibody dependent cellular cytotoxicity (ADCC) and risk of HIV infection [2,5]. The possibility that non-neutralizing ADCC competent Abs may be implicated in preventing HIV infection provides a rationale for quantifying non-neutralizing Abs (NnAbs) endowed with Fc mediated effector functions in HIV infected individuals [6,7].

HIV Env glycoprotein is the HIV gene product targeted by ADCC since it is the only viral protein expressed on the surface of infected cells [8]. HIV Env exposed on HIV virions and on the surface of infected cells are highly glycosylated spikes, composed of a heterotrimer of the surface glycoprotein gp120 non-covalently associated with the transmembrane glycoprotein gp41 [9,10,11,12,13,14]. Native Env is present in a “closed” conformation on the surface of infected cells [15]. This native conformation can be recognized by BnAbs and some NnAbs to mediate Fc-dependent effector functions such as ADCC. Env interactions with CD4 drive the transition of the closed Env conformation to a CD4 bound “open” conformation [15,16]. The HIV Env open conformation is normally absent from the surface of productively infected cells since CD4 is downregulated by HIV Nef and/or Vpu [17,18,19]. However, gp120/CD4 conjugates can transiently occur during viral entry, when the virion binds to CD4^+^ cells during infection, but also on the surface of uninfected CD4^+^ bystander cells [20]. Indeed, Env trimers are not stable. Consequently, productively infected cells shed gp120, which is taken up by the cell surface CD4 on uninfected bystander cells exposing CD4 induced epitopes normally hidden inside Env trimers [20]. These epitopes are recognized by monoclonal Abs such as A32 and C11 specific for a highly-conserved cluster A region, making uninfected bystander cells susceptible to ADCC mediated by these Abs [20,21]. BnAbs bind to epitopes other than those in the cluster A region and can mediate ADCC, but are rare in plasma from HIV infected individuals [22,23,24].

The amount and specificity of anti-Env Abs to the open or closed Env conformation in plasma samples are critical parameters, which most likely impact directly on their ADCC competence. Several assays have been used to quantify ADCC activity to target cells expressing HIV Env [20]. Among these are the ADCC-GranToxiLux assay, which measures the delivery of granzyme B to target cells, an early step in the pathway leading to target cell apoptosis [25], diverse assays that measure the elimination of target cells [26,27], and the Rapid Fluorescence ADCC assay [28], which does not measure ADCC activity, but rather trogocytosis, as defined by the transfer of target cell membranes to effector cells [29]. The target cells used in these assays are either recombinant gp120 (rgp120) coated CEM.NKr.CCR5 (CEM) cells [25,30,31,32,33,34], HIV infected CEM or primary CD4^+^ T cells [20,22,23,24]. The rgp120 used to coat CEM cells, like gp120 shed by infected cells, has a conformation that is distinct from native trimeric Env on target cells infected with wild type HIV. The use of rgp120 coated or HIV-infected cells as ADCC target cells using assays that do not distinguish infected from uninfected bystander cells has led to the widely held view that the only ADCC-competent Abs present in plasma from HIV^+^ individuals are specific for cluster A CD4 induced Env epitopes [17,18,20,30]. 

Here, we developed two new flow cytometry-based methods to quantify the levels of Env-specific Abs in HIV^+^ plasma. One method used rgp120 coated CEM (cCEM) cells and the other used HIV-infected CEM cells selected for being HIV^+^ (iCEMs). Quantification of Env-specific Abs in HIV^+^ plasma using these two methods were compared to results generated using a previously described rgp120 coated enzyme linked immunosorbent assay (ELISA) [35]. The rgp120 used to coat ELISA plates and on cCEM was present in an open conformation exposing CD4 induced epitopes. ICEMs expressed Env in a closed conformation and had downmodulated their cell surface CD4 expression. Using iCEM cells allowed us probe HIV^+^ plasma for the presence of Abs to closed conformation Env exposing no CD4 induced epitopes. We quantified the levels of anti-gp120/HIV Env Abs in plasma from 78 HIV-infected individuals, including untreated progressors, individuals successfully treated with anti-retroviral therapy, and elite controllers using these three methods. We showed that Env-specific Abs in HIV^+^ plasma samples preferentially recognized monomeric-linear epitopes, including CD4 induced epitopes. However, because HIV^+^ plasma also bound iCEM cells, we showed, for the first time, that HIV^+^ plasma also contains Abs to native Env epitopes. There was a positive correlation between the amount Env-specific Abs measured in plasma samples using these three methods. By comparing subject groups, we showed that plasma from treated progressors with undetectable viral loads (VL) had lower Env-specific Ab levels than untreated progressors and elite controllers did. Untreated progressor and elite controller plasma had similar levels of Env-specific Abs despite elite controllers having undetectable VLs.

## 2. Materials and Methods

### 2.1. Ethics Statement

This study was approved by the Institutional Review Boards of the Comité d’Éthique de la Recherche du Centre Hospitalier de l’Université de Montréal, project identification code, 17-096, July 2018 and of the McGill University Health Centre, project identification code 2018-4505, July 2018. Informed consent was obtained from all study participants. 

### 2.2. Study Subjects

For this study, we used plasma samples from 3 groups of HIV-infected individuals in the chronic phase of HIV infection. Untreated progressors (*n* = 18) had CD4 counts <400 cells/mL and a VL of >10,000 HIV RNA copies/mL of plasma, treated progressors (*n* = 24) had CD4 counts <400 cells/mL and a VL of <50 copies/mL of plasma and elite controllers (*n* = 37) had CD4 counts >400 cells/mL and an HIV VL of <50 copies/mL of plasma. The untreated and treated progressors were drawn from subjects enrolled in the Montreal Primary Infection Cohort. These samples were from time points collected at least 1 year post infection. Those from treated progressors were from persons receiving antiretroviral therapy that controlled HIV VL for at least 1 year. The elite controller samples were drawn from participants in the Canadian Cohort of HIV-Infected Slow Progressors [36].

### 2.3. Gp120 Capture Plate-Based ELISA

The gp120-capture plate-based ELISA has been described elsewhere [35]. Briefly, ELISA plates (Nunc MaxisSorp, Thermo Fisher Scientific, Whitby, ON, Canada) were coated with 2.5 µg/mL D7324, a sheep anti-gp120-specific capture Ab (Aalto Bio Reagents, Dublin, Ireland) in 0.037 M Na_2_CO_3_ buffer, pH 9.5 (coating buffer) overnight at 4 °C. Plates were washed 3 times with phosphate buffered saline (PBS, Wisent Bio Products, St-Jean-Baptist, QC, Canada); 0.05% Tween 20 (Sigma-Aldrich, St. Louis, MO, USA) (PBST, wash buffer) and blocked with PBS; 0.05% Tween 20; 1% bovine serum albumin (Sigma-Aldrich) (blocking buffer) for 30 min at 37 °C in a humidified, 5% CO_2_ incubator. One hundred μL of HIV-1 rgp120 (from the NIH Reagent Bank, HIV-1 BaL gp120 recombinant protein from DAIDS, NIAID) at 100 ng/mL in PBST was added to each well of a 96-well plate for 3 h at room temperature (RT). The following additions were made to the ELISA plates, washing 3 times with wash buffer between steps. 100 μL/well of diluted plasma, positive and negative controls were added to each well in duplicate for 1 h at 37 °C in a humidified 5% CO_2_ incubator. Plasma from each study subject was serially two-fold diluted in blocking buffer starting at a dilution of 1:100. The positive control was anti-HIV Immunoglobulin (HIVIG, a pool of polyclonal IgG isolated from HIV-infected donors from the NIH Reagent Bank, NABI and NHLBI). HIVIG was serially three-fold diluted starting at 150 μg/mL. Wells with no rgp120 served as a negative control. Binding of anti-gp120 specific Abs to rgp120 was detected by adding 100 μL per well of horseradish peroxidase conjugated-goat anti-human IgG Fc secondary Ab diluted 1:7500 in blocking buffer (Invitrogen, Frederick, MD, USA) for 30 min at RT. Binding of the secondary Ab was detected by adding 100 μL/well of 3,3’,5,5’-tetramethylbenzidine (TMB) substrate (Thermo Fisher Scientific) until the desired color development was achieved. The reaction was stopped by adding 100 μL of 1M phosphoric acid (H_3_PO_4_, Thermo Fisher Scientific). Plates were read at an optical density of 450 nm on an ELISA microplate reader (Infinite 2000 PRO, Tecan Group Ltd., Männedorf, Switzerland). The concentrations of the anti-gp120 specific Abs in each plasma samples were obtained by interpolating from the HIVIG standard curve using GraphPad Prism version 7.00 (GraphPad Software, La Jolla, CA, USA). Only values that fell within the linear range of the standard curve were used to calculate anti-gp120 specific IgG plasma concentrations in μg/mL relative to HIVIG. 

### 2.4. Flow Cytometry-Based Env-Specific Ab Quantification Assay Using rgp120 Coated CEM (cCEM) Cells

In this assay, cCEM cells were used as target cells. They were prepared by incubating 1 × 10^6^ CEM cells with 0.6 µg of the same rgp120 as the one used to coat ELISA plates in Section 2.3, in 100 µL of RPMI 1640; 10% fetal bovine serum (FBS); 2 mM l-glutamine; 100 IU/mL penicillin; 100 mg/mL streptomycin (R10) (all from Wisent) at 37 °C in a 5% CO_2_ humidified incubator for 75 min. Cells were washed twice and resuspended to 4 × 10^6^ CEM cells per ml in PBS: 4% FBS. Uncoated CEM cells served as an internal negative control. They were distinguished from target cells by staining with carboxyfluorescein succinimidyl ester (CFSE, Life Technologies, Burlington, ON, Canada). Briefly, CEM cells, at 2 × 10^6^ cells/mL of PBS, were mixed with 1 mL of 0.32 μM CFSE and incubated for 8 to 10 min at RT. The reaction was stopped by adding 1 mL of FBS at RT for 8 to 10 min. The cells were washed and resuspended at 4 × 10^6^ cells/mL in PBS; 4% FBS. CCEM and CFSE^+^ CEM cells were mixed at a ratio of 1:1 and 25 µL containing 5 × 10^4^ rgp120-coated CEM cells and 5 × 10^4^ CEM cells were plated into each well of a V-bottomed 96-well plate in duplicate (Sarstedt Inc., Montreal, QC, Canada). Serial 3-fold dilutions of HIVIG, starting at a concentration 150 μg/mL, was used to generate a standard curve. Twenty-five µL of diluted plasma or HIVIG were added to wells containing cCEM and CEM cells and incubated for 20 min at RT in the dark. Cells were washed twice with 100 µL of PBS: 4% FBS. Bound Abs were detected by adding 50 μL of a 1:50 dilution of an APC-conjugated anti-human IgG Fc (huIgGFc, BioLegend, Burlington, ON, Canada) to each well for 20 min at 4 °C in the dark. Plates were washed twice with PBS; 4% FBS and fixed with 2% paraformaldehyde (Santa Cruz Biotechnology, Dallas, TX, USA). At least 30,000 cells were acquired from each well of the 96-well plates using an LSR Fortessa X20 instrument (BD Biosciences, Mississauga, ON, Canada) and a high-throughput system. The results were analyzed using FlowJo software version 10 (Tree Star, Inc., Ashland, OR, USA). Negative controls included binding to CFSE^+^ CEM cells present in the same well as the unlabelled cCEM cells and a no Ab control included in the same plate. 

### 2.5. Preparation of HIV-Infected CEM (iCEM) Cells

ICEM cells were generated by infecting CEM cells with the replication competent NL4-3-Bal-IRES-HSA construct and sorting for cells expressing heat stable antigen (HSA) also known as murine CD24. The NL4-3-Bal-IRES-HSA viral construct was a kind gift from Dr. Michel Tremblay (Laval University, Quebec, QC, Canada) [37]. CEM cells were HIV-infected by adding supernatant from NL4-3-Bal-IRES-HSA transfected 293T cells to 10^6^ CEM cells followed by spinoculation at 2000× *g* for 90 min. Cells were then incubated for 30 min at 37 °C in a humidified 5% CO_2_ incubator before washing twice with R10. Cell surface expression of HSA was used to identify HIV-infected cells. On average 52% were HSA^+^ (range 45 to 73%) four days post infection. To isolate the iCEM from uninfected CEM cells, we stained them with PECy7-conjugated rat anti-mouse CD24 specific monoclonal Ab (Clone M1/69, BD Biosciences) and sorted for cells expressing HSA using a FACSAria instrument (BD Biosciences). To confirm that cells were HIV infected, sorted, expanded iCEM cells were stained for cell surface CD4 with BV421-conjugated anti-human CD4 mAb (Clone OKT4, BioLegend), cell surface HSA expression with PECy7-conjugated anti-mouse CD24 and intracellularly for HIV p24 using FITC-conjugated anti-p24 (Clone KC57, Beckman Coulter, Mississauga, ON, Canada). To confirm cell surface HIV Env expression, we stained sorted iCEM cells with the BnAb 2G12 monoclonal Ab (from the NIH AIDS Reagent Program, Division of AIDS, NIAID, NIH: Anti-HIV-1 gp120 monoclonal Ab 2G12 from Dr. Hermann Katinger [38,39,40,41,42]) and the NnAb A32 monoclonal Ab (from the NIH AIDS Reagent Program, Division of AIDS, NIAID, NIH: HIV-1 gp120 monoclonal Ab A32 from Dr. James E. Robinson [43,44]) for 20 min at RT. 2G12 binds both closed and open conformation Env at a CD4 independent outer domain epitope [39]. A32 is specific for a CD4 induced epitope on open conformation Env. Cells were washed with 100 µL of PBS; 4% FBS and stained with APC-conjugated anti-huIgGFc, (BioLegend). Stained cells were fixed in 2% paraformaldehyde (Santa Cruz). At least 30,000 cells were acquired using an LSR Fortessa X20 flow cytometer instrument. CEM cells were also stained and acquired in parallel to quantify non-specific background binding. 

### 2.6. Flow Cytometry-Based Env-Specific Ab Quantification Assay Using iCEM

In this assay, iCEM were used as target cells. Non-specific binding to CEM cells was measured simultaneously in the same wells. CEM cells were distinguished from iCEM cells by staining them with CFSE as described above. iCEM and CFSE^+^ CEM cells were mixed at a ratio of 1:1 and plated by adding 25 μL of cells to each well of V-bottomed 96-well plates in duplicate (Sarstedt Inc.). Plasma samples were serially 3-fold diluted starting at dilutions of either 1:10 for treated progressors or 1:100 for untreated progressors and elite controllers in cold PBS; 4% FBS. Serial 3-fold dilutions of HIVIG, starting at a concentration 150 μg/mL, was used to generate a standard curve. Twenty-five µL of plasma or HIVIG dilutions were added to the cells for 20 min at RT in the dark. Each plate had an internal no Ab negative control. After the Ab incubation step, plates were washed twice with 100 μL/well of PBS; 4% FBS. Ab binding was detected by adding 50 μL of 1:50 dilution of APC-conjugated anti-huIgGFc (BioLegend) to each well for 20 min at 4 °C in the dark. Plates were then washed twice with PBS; 4% FBS and fixed with 2% paraformaldehyde (Santa Cruz). At least 30,000 cells were acquired from each well of the 96-well plates using an LSR Fortessa X20 instrument and a high throughput system. The results were analyzed using FlowJo software version 10 (Tree Star, Inc.). Negative controls included binding to CFSE^+^ CEM cells in the same well and a no Ab control in the same plate.

### 2.7. Data Analysis

Abs from study subjects binding to rgp120 on ELISA plates and to cCEM as well as to Env on iCEM cells were quantified by interpolating from the HIVIG standard curve present in the same 96-well plates as the test samples. Values that lay within the linear range of the standard curve were selected to calculate Ab concentrations. When values for 2 or more sample dilutions were within the standard curve’s linear range, the mean of these results was used to assign the anti-Env Ab concentration for that sample. In the rgp120 plate-based ELISA assay, values generated by Ab binding were background subtracted by the values generated when no Ab was present. For the flow cytometry-based assays, each 96-well plate included a no Ab negative control that evaluated binding generated by the anti-huIgGFc secondary Ab. Values generated in these wells were subtracted from those of the test wells. CFSE^+^ CEM served as a within-well internal negative controls measuring non-HIV Env-specific binding. Binding levels to CEM were subtracted from results generated by the same Ab dilution used to assess binding to cCEM and iCEM cells. HIVIG is a 50 mg/mL protein solution of purified Ab. The same HIVIG preparations was used for all assays permitting comparison between assays. However, the amount of anti-gp120 specific Abs in the polyclonal HIVIG solution is unknown. For this reason, the quantity of the Abs measured for all subjects were reported as arbitrary units (AU) per mL of plasma. 

The average intra-assay coefficient of variation and (95% confidence intervals) for the HIV^+^ plasma samples tested in duplicate in the plate-based ELISA was 4.03% (3.77, 4.92). The average inter-assay coefficient of variation for HIVIG standard curves run 8 times in the plate-based ELISA was 15.18% (12.81, 17.55). The average intra-assay coefficient of variation for HIV^+^ plasma sample duplicates was 2.87% (0.77, 4.97) for flow cytometry quantification experiments using cCEM and 4.57% (3.07, 6.07) for those using iCEM cells. The average intra-assay coefficient of variation for 5 HIVIG standard curves using cCEM as target cells was 7.96% (5.86, 10.06) and for the 8 HIVIG standard curves using iCEM at target cells was 7.74% (5.74, 9.74). Plasma from 7 individuals were tested on two occasion. The average inter-assay coefficient of variation for experiments using cCEM and iCEM target cells was 15.9% (13.8, 18) and 11.5% (9.4, 13.6) respectively. 

### 2.8. Statistical Analysis

GraphPad Prism version 7.00 or 8.00 for Windows, (GraphPad Software, Inc., La Jolla, CA, USA) was used for statistical analyses and graphical presentation. The significance of between-group differences in monoclonal Ab binding to CEM, cCEM and iCEM as well as for AU results for untreated progressors, treated progressors, and elite controllers was assessed using Kruskal-Wallis tests with Dunn’s post tests. The significance of within-individual differences in AUs generated using the three methods were assessed using Friedman tests with Dunn’s post tests. The significance of the correlation between results obtained using the plate-based ELISA assay, and the two flow cytometry based binding assays was assessed using Spearman’s correlation tests. 

## 3. Results

### 3.1. Characterization of iCEM Cells

Plate-based ELISA methods that quantify gp120 specific Abs in plasma from HIV infected persons detect Abs to linear gp120 epitopes including CD4 induced epitopes that are normally hidden in native trimeric Env expressed on the surface of cells infected with wild type HIV. HIV infected cell cultures include not only infected cells but also uninfected CD4^+^ bystander cells [20]. The CD4 on bystander cells interacts with gp120 shed from infected cells and/or HIV virions originating from the infecting inoculum [20,45]. Consequently, anti-gp120 Abs in HIV^+^ plasma preferentially bind CD4 induced epitopes on uninfected bystander cells. This situation precludes identifying the contribution of Abs to native closed Env on HIV infected cells versus Abs to open Env on bystander cells in plasma from HIV^+^ subjects. Therefore, to measure the binding of Env-specific Abs in plasma from HIV^+^ individuals to a closed conformation of trimeric Env expressed on HIV-infected cells, we generated iCEM cells expressing native trimeric Env. 

**Figure 1:**
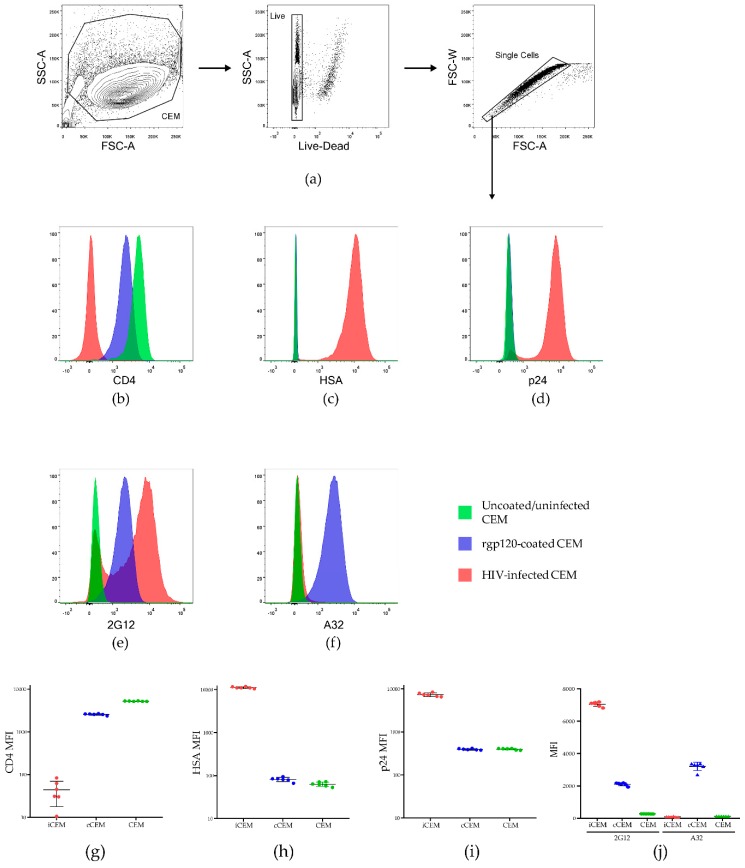
Characterization of HIV infected CEM (iCEM) cells. ICEM and CFSE^+^ CEM cells were stained with a panel of monoclonal antibodies to cell surface CD4, HSA, intracellular p24, and cell surface 2G12 and A32. Live singlet cells were gated on (**a**). Histograms show expression of (**b**) CD4, (**c**) HSA, (**d**) intracellular p24, (**e**) the HIV Envelope epitope detected by 2G12 and (**f**) the CD4 induced epitope detected by A32 on CEM cells (in green) cCEM cells (in blue) and iCEM (in pink). The MFI of CD4^+^ (**g**), HSA^+^ (**h**) and p24^+^ (**i**) CEM, cCEM, and iCEM cells. The mean fluorescence intensity of 2G12 and A32 staining to CEM, cCEM, and iCEM cells (**j**). FSC-A = forward scatter-area; SSC-A = side scatter-area; FSC-W forward scatter width; HSA = heat stable antigen, also known as murine CD24; MFI = mean fluorescence intensity.

Figure 1 shows the results of staining live singlet CEM, cCEM and iCEM with monoclonal Abs to CD4, HSA, intracellular p24, 2G12 and A32. Figure 1a shows the strategy for gating on live singlet cells. Figure 1b–f show examples of staining CEM, cCEM, and iCEM cells with these five monoclonal Abs. Figure 1g–j show the results generated for staining six replicates of CEM, cCEM and iCEM cells with these monoclonal Abs. CD4 was expressed on a mean ± standard deviation of 99.5 ± 0.24%, 97.9 ± 0.41% and 0.47 ± 0.01% of CEM, cCEM, and iCEM cells, respectively. HSA was detected on 0.32 ± 0.3%, 0.54 ± 0.21% and 99.6 ± 0.2% of CEM, cCEM, and iCEM cells. Intracellular p24 was present in 0.52 ± 0.16, 0.53 ± 0.17% and 95.66 ± 0.6% of CEM, cCEM, and iCEM. Thus, CD4 was downmodulated on iCEM, likely due to the actions of HIV Nef and Vpu making CD4 unavailable to interact with Env on these cells. HIV infection of iCEM cells was confirmed by the expression of the HSA selection marker encoded by the HIV viral isolate they were infected with and the presence of intracellular p24.

Monoclonal Abs 2G12 and A32 bound CEM cells at background levels. 2G12 and A32 bound 0.91 ± 0.8% and 0.27 ± 0.3%, of CEM cells with a mean fluorescence intensity of 272 ± 5 and 119 ± 1.6, respectively. 2G12 bound 96.07 ± 0.34% and 80.42 ± 0.86% of cCEM and iCEM cells with a lower mean fluorescence intensity for cCEM than for iCEM recognition (2105 ± 92 versus 7049 ± 141 for cCEM and iCEM cells) though these differences did not achieve statistical significance (Kruskall-Wallis test with Dunn’s post test. A32 bound a higher frequency of cCEM than iCEM cells (97.85 ± 0.77% versus 2.3 ± 0.3% *p* < 0.001, Dunn’s post test). The mean fluorescence intensity of A32 binding to cCEM cells was also higher than that to iCEM cells (3209 ± 257 versus 89.2 ± 21, *p* < 0.001, Dunn’s post test). The mean fluorescence intensity of A32 binding to iCEM was as low as that to CEM cells (*p* > 0.05, Dunn’s post test). In summary, 2G12 detected a non-conformation dependent HIV Env epitope present on both cCEM and iCEM cells. Monoclonal Ab A32 detected a CD4 induced epitope only on cCEM cells. The low level of A32 binding to iCEM is consistent with CD4 induced epitopes not being exposed on Env expressed on iCEM cells, supporting the conclusion that Env is in a closed conformation on these cells.

### 3.2. Flow Cytometry-Based Env-Specific Ab Quantification Assay 

**Figure 2:**
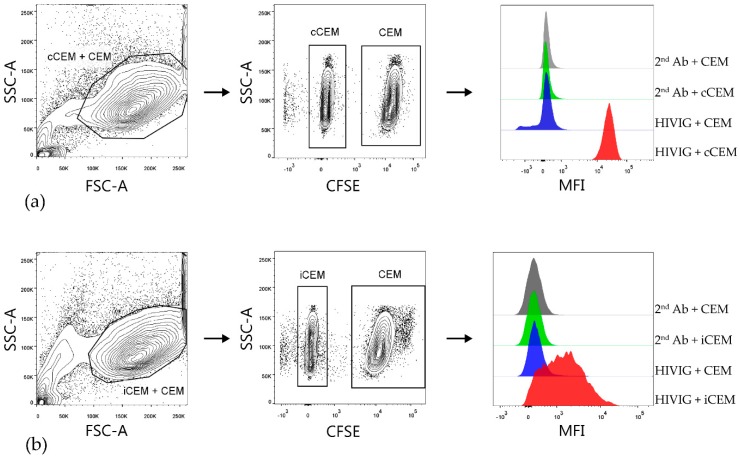
Gating strategy used to detect HIVIG binding to cCEM and iCEM cells. Both cCEM and CFSE^+^ CEM (left panel of (**a**)) or iCEM and CFSE^+^ CEM cells (left panel of (**b**)) were gated on. From these, cCEM and iCEM were distinguished from CFSE^+^ CEM cells (middle panels of (**a**) and (**b**) respectively). Binding of secondary antibody specific for human IgG Fc to CEM and cCEM (1st and 2nd rows of right panel of (**a**)) or CEM and iCEM (1st and 2nd rows of right panels of (**b**)). Binding of HIVIG primary antibody at 150 μg/mL to CEM and cCEM (3^rd^ and 4^th^ rows of right panel of (**a**)) and CEM and iCEM (3rd and 4th rows of right panel of (**b**)) was detected by using a fluorochrome conjugated secondary Ab. FCS-A = forward scatter-area; SSC-A = side scatter-area; CFSE = carboxyfluorescein succinimidyl ester; CEM = CEM.NKr.CCR5; cCEM = recombinant gp120 coated CEM cells; iCEM = HIV infected CEM cells; MFI = mean fluorescence intensity. 2nd Ab = anti-human immunoglobulin G Fc specific secondary antibody; Fc = the fragment crystallizable portion of immunoglobulin G.

In order to quantify and compare the relative amounts of HIV Env-specific Abs in plasma from HIV^+^ subjects, we developed two flow cytometry-based Ab binding assays using either cCEM or iCEM as target cells. After gating on cCEM+ CEM (Figure 2a) cells and iCEM+ CEM (Figure 2b, left-hand panels), cCEM, and iCEM cells were distinguished from CFSE^+^ CEM by flow cytometry (Figure 2, middle panels). Figure 2, right hand panels show from the top to the bottom rows the binding of secondary Ab to CFSE^+^ CEM and CFSE^-^ cCEM and binding of HIVIG to CFSE^+^ CEM and CFSE^-^ cCEM cells (a) and the same for binding of secondary Ab and HIVIG to CFSE^+^ CEM and CFSE^-^ iCEM cells (b). HIVIG bound to both cCEM and iCEM cells with a higher mean fluorescence intensity than to their internal negative controls. Secondary Ab recognized CFSE^-^ cCEM and iCEM and CFSE^+^ CEM with equivalent, low mean fluorescence intensities. 

**Figure 3:**
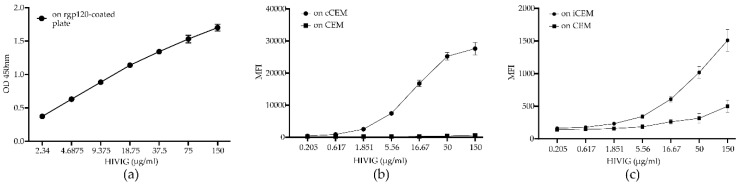
Standard curves generated by binding HIVIG to plates coated with recombinant gp120, recombinant gp120 coated CEM (cCEM) cells and to HIV-infected CEM (iCEM) cells. Binding of a 2-fold serial dilution of HIVIG to ELISA plates coated with rgp120 (**a**). Binding of a 3-fold serial dilution of HIVIG to cCEM (**b**) and iCEM (**c**) and their CFSE^+^ CEM cell internal controls. The y-axis shows the optical density measured at 450 nm (OD450nm) generated by HIVIG binding to rgp120 coated plates (**a**). In (**b**) and (**c**), the y-axes show the mean fluorescence intensity (MFI) generated by HIVIG binding to (**b**) cCEM (closed circles) and CEM (closed squares) and (**c**) to iCEM (closed circles) and CEM (closed squares). The standard curve in (**b**) shows average values for 5 replicates; the curve in (**c**) is shows average values for 8 replicates. Each point and its error bars represent averages and standard deviations for these values. OD450nm = optical density at a wave length of 450 nanometers; MFI = mean fluorescence intensity; CEM = CEM.NKr.CCR5 cell line.

Figure 3 shows the standard curves generated by HIVIG binding to rgp120 coated ELISA plates (a), cCEM and CFSE^+^ CEM (b) and to iCEM and CFSE^+^ CEM cells (c). HIVIG recognized cCEM and iCEM cells with a higher mean fluorescence intensity than CEM cells. These results show that the mean fluorescence intensity of HIVIG binding to cCEM was higher than to iCEM cells. 

### 3.3. HIV Env-Specific Ab Quantification in Plasma Samples from HIV^+^ Subjects

**Figure 4:**
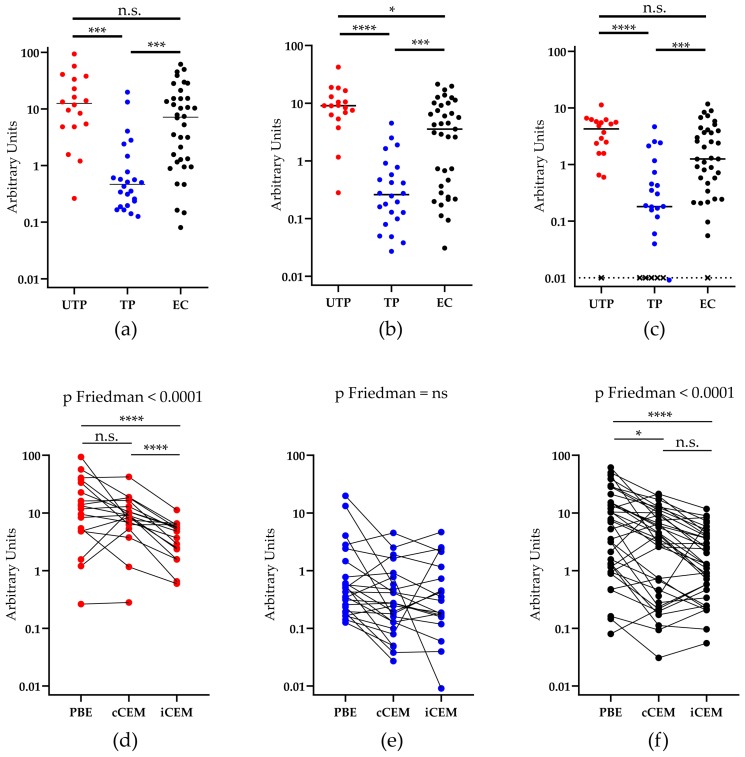
Quantification of antibodies to rgp120/HIV Envelope-using three methods. The y-axis shows the relative amount of recombinant gp120 or HIV Envelope-specific antibody measured in plasma from three HIV^+^ subject groups using (**a**) a plate-based ELISA assay, or by flow cytometry-based assays using (**b**) cCEM and (**c**) iCEM cells as target cells. The subject groups being compared are indicated by lines joining two groups and the significance of between-group differences is indicated by “*” symbols over the lines joining the two groups being compared. Anti-rgp120/HIV Envelope-specific antibody levels in 1 untreated progressor, 5 treated progressors and 1 elite controller were below the limit of quantitation when iCEM cells were used as target cells and are represented by an “×” (**c**). Plasma from (**d**) untreated progressors, (**e**) treated ‘progressors, and (**f**) elite controllers were tested for their capacity to bind rgp120 coated wells in the plate-based ELISA assay to cCEM and to iCEM cells. PBE = plate-based ELISA; UTP = untreated progressors; TP = treated progressors; EC = elite controllers; “*” = *p* < 0.05; “***” = *p* < 0.001, “****” = *p* <0.0001, n.s. = not significant.

We questioned whether Abs in plasma from untreated progressors, treated progressors and elite controllers differed in their ability to bind plate-bound rgp120, cCEM, and iCEM cells. Our results showed that plasma from treated progressors, as compared to those from untreated progressors and elite controllers, contained significantly lower levels of Abs to plate-bound rgp120, cCEM and iCEM cells (Figure 4a–c, Kruskal-Wallis test with Dunn’s post-tests). Plasma from 1 untreated progressor, 5 treated progressors, and 1 elite controller bound iCEM at levels below the detection limit (Figure 4c). Since, we did not detect any Ab binding to iCEM cells by plasma from these subjects, they were excluded from further analyses. Plasma from untreated progressors had higher levels of Env-specific Abs than plasma from elite controllers, but this difference only achieved statistical significance for Abs recognizing cCEM cells (Kruskal-Wallis tests with Dunn’s post-tests (Figure 4a–c). 

The binding results were re-analyzed by examining how plasma from each study subject bound Env in the three Ab quantification assays. We found that within-subject differences in the ability of plasma Abs from treated progressors to recognize rgp120 coated ELISA plates, cCEM and iCEM cells did not differ significantly (Figure 4e, *p* > 0.05, Friedman test). Plasma from untreated progressors bound the linear rgp120 on coated plates and on cCEM at levels that were not significantly different from each other, but were at higher levels than to iCEM (Figure 4d, *p* < 0.0001 for both, Dunn’s post tests). Plasma from elite controllers bound rgp120 coated plates at a higher level than they bound cCEM and iCEM cells (Figure 4f, *p* < 0.05 and *p* < 0.0001, respectively, Dunn’s post tests). The binding levels of plasma from elite controllers to cCEM and iCEM did not differ significantly. 

**Figure 5:**
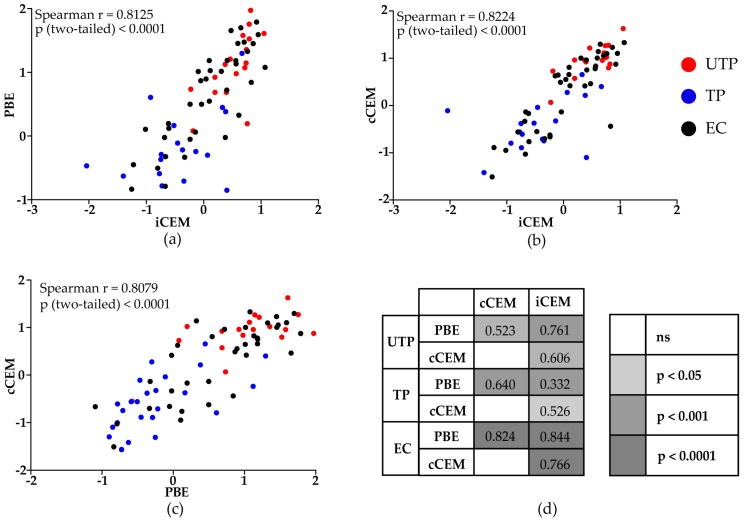
Correlations between rgp120/HIV Envelope-specific antibody levels quantified by a plate-based ELISA and two flow-cytometry based assays. Spearman correlation tests were used to evaluate the significance of the correlation between results generated by the (**a**) plate-based ELISA and flow cytometry-based quantification assays using iCEM as target cells, (**b**) the two flow cytometry-based quantification assays using iCEM and cCEM cells as target cells, and (**c**) the plate-based ELISA assay and the flow cytometry-based quantification assays using cCEM as target cells for all HIV-infected subjects (**a**–**c**) or for untreated progressors, treated progressors and elite controllers separately (**d**). Values in (**d**) indicate the correlation coefficient “r” for each comparison. The color scale indicates the “p” values for each correlation. PBE = plate-based ELISA; cCEM = recombinant gp120 coated CEM cells; iCEM = HIV-infected CEM cells; UTP = untreated progressors; TP = treated progressors; EC = elite controllers.

The 3 methods generated results that were correlated with each other when all study subjects were considered, (Figure 5a–c, *r* > 0.80, *p* < 0.0001, Spearman correlation tests). Results generated by the three assays were significantly positively correlated for untreated progressors, treated progressors and elite controllers (Figure 5d). 

In summary, these results indicate that Env-specific Abs in HIV^+^ plasma samples preferentially targeted the CD4 induced epitope exposed on the open Env conformation, which is exposed on the linear rgp120 used to coat ELISA plates and on cCEM cells. However, there exists a subset of Env-specific Abs in HIV^+^ plasma that recognize Env in its closed conformation as shown by their ability to bind iCEM cells. Untreated progressors have higher levels of anti-Env-specific Abs than do treated progressors. This suggests that antigenemia, which in HIV^+^ persons is represented by detectable HIV VL, drives and maintains high levels of Env-specific Abs. High levels of Env-specific Abs without detectable VL is a distinctive characteristic of elite controllers possibly associated with the maintenance of a strong memory B cells compartment in these individuals [46,47].

## 4. Discussion

In this report, we describe two new flow cytometry-based assays that quantitate Abs specific for HIV Env by interpolation from an HIVIG standard curve. One method recognizes cCEM as a target cell and the other iCEM cells. Anti-HIV Env-specific Abs in plasma from HIV-infected untreated progressors, treated progressors and elite controllers were compared for their ability to recognize Env in the two flow-cytometry based assays and in a plate-based ELISA assay in which wells were coated with rgp120. Plasma from untreated progressors and elite controllers had higher levels of anti-gp120-specific Abs in all three assays compared to plasma from treated progressors. The concentration of plasma IgG in µg/mL from each study subject binding HIV Env in these three assays was significantly correlated for untreated progressors, treated progressors and elite controllers. 

Native Env is a trimer assembled of heterodimers made up of gp120 and gp41 glycoproteins. While gp120 forms the outer part of the trimer, gp41 is mostly buried at the trimer interface and anchors Env into the plasma membrane [9,10,11,12]. Env interactions with CD4 drive the transition from a closed Env conformation to a CD4 bound open conformation [15,16]. CD4 is downregulated from the surface of productively infected cells by Nef and Vpu [17,18,19]. Unliganded Env is normally present in a closed conformation on HIV-infected cells [15]. Advancements in electron microscopy and cryo-tomography have shown that highly conserved epitopes are hidden in the native Env trimer [13,48,49,50,51]. 

The iCEM cells, used as anti-HIV Env binding targets, were 99.6 ± 0.2% HSA^+,^ and 95.66 ± 0.6% intracellular p24^+^. Less than 1% expressed CD4 at a low mean fluorescence intensity. Staining with monoclonal Ab 2G12 confirmed that 80.42 ± 0.86% expressed HIV Env at a mean fluorescence intensity of 7049 ± 141, which was 26-fold over that to CEM cells. The low frequency and intensity of staining by monoclonal Ab A32 to iCEM cells indicated that Env on these cells maintains a closed conformation. On the other hand, rgp120 used to coat ELISA plates and Env present on the surface of cCEM is monomeric, linear and recognized by the NnAb A32 specific for a CD4 induced epitope only exposed on Env in an open conformation. Plasma from HIV^+^ subjects readily recognized epitopes on rgp120 coated plates and on cCEM cells. On the other hand, iCEM cells are highly enriched for the presentation of closed conformation HIV Env, the conformation which is present on wild type HIV infected cells. They thus have a superior capacity than do HIV infected and bystander cells present in recently HIV infected cultures to bind Abs in HIV+ plasma to closed conformation Env [20,45]. Using iCEM cells as target cells overcomes problems inherent in interpreting results of anti-Env Ab binding using recently infected CD4 cells to probe HIV^+^ plasma for the presence of Abs specific for Env on productively infected cells. Using iCEM cells, we were able to confirm that HIV^+^ plasma contains Abs recognizing closed conformation HIV Env. These iCEM cells will be useful as target cells for ADCC assays. They can be used to assess whether Abs to closed conformation Env can indeed target and kill productively HIV infected cells and not just bystander cells 

Others have also described methods to detect gp120- or Env-specific Abs in HIV^+^ plasma [35,52,53]. Two of these methods were used to detect Abs in HIV^+^ plasma specific for monomeric linear HIV gp120 [35,52]. Veillette et al. reported detecting Env in a 3-dimensional conformation on the surface of transfected cells [53]. However, the method used provided a relative quantification since it did not use a standard curve with a known source of Env-specific Abs to interpolate results from plasma samples. Furthermore, the preparation of target cells for this assay relied on antigen availability, which was associated with transfection efficiency. In other words, not all the target cells used to probe for Env-specific Abs expressed Env. Furthermore, between-preparations differences in transfection efficiency may compromise the ability to compare results generated using different batches of transfected Env expressing cells. The use of iCEMs, which are essentially all positive for cell surface Env overcomes the limitations of these assays by only expressing Env in a closed conformation and by eliminating concerns relating to inter-batch variability due to transfection efficiency.

We observed that plasma from treated progressors had lower concentrations of Env-specific Abs in all three assays. This is probably due to antiretroviral therapy dependent reduction in HIV VL. Presence of antigen is likely needed to maintain HIV Env-specific Ab responses. HIV-specific T cell responses also decline drastically after the initiation of antiretroviral therapy [54]. Results generated by the three assays using plasma from the three study populations were correlated. Untreated progressors have an uncontrolled HIV VL, which drives persistent anti-HIV Env Ab responses. Interestingly, elite controllers, who have undetectable HIV VLs maintain robust anti-Env specific Ab responses. The reason for this is unclear. One possibility is that elite controllers maintain a strong memory B-cell response, which may also be involved in VL control [46,47]. Even though elite controllers have VLs < 50 copies/mL of plasma, there is evidence they have HIV VLs below this detection limit and low-level viral replication [55,56,57]. This could potentially explain the maintenance of high anti-Env-specific Ab levels in the setting of the VL suppression seen in elite controllers.

Polyclonal IgG from HIV^+^ individuals, was used to generate a standard curve for all three assays used to quantify anti-Env specific Abs. By interpolating results from the three assays with the same range of HIVIG concentrations it was possible to compare results from all three assays and to confirm that plasma from HIV^+^ individuals include Abs to both linear and 3-dimensional Env epitopes. The use of internal negative controls in the form of CEM cells permitted detection of Abs to these target cells that were not Env-specific. The exact amount of anti-gp120 or anti-Env specific Abs in HIVIG is not precisely known, though we have estimated that anti-gp120 specific Abs represent approximately 5% of the total IgG pool. This is the reason that quantification of results in relation to HIVIG concentrations were defined as AUs rather than concentrations of anti-gp120-specific Abs.

Generation of Abs that bind and neutralize a broad range of HIV isolates is one of the major goals of current HIV vaccine strategies. But there are significant obstacles to achieving this goal [58,59,60,61,62]. Results from the RV144 HIV vaccine trial, simian immunodeficiency virus infected (SIV) rhesus macaque studies, and in HIV elite controller studies have shown that there is a significant proportion of Env-specific Abs in plasma from vaccinees, rhesus macaques and elite controllers that mediate non-neutralizing functions such as ADCC [2,63,64,65]. These Abs have been implicated in HIV/SIV protection and control [64]. HIV elite controllers represent a unique example of a functional cure as they control HIV without antiretroviral therapy. While cellular immunity is certainly important in elite controller HIV suppression, elite controllers also generate Abs with unique signatures that perform non-neutralizing functions [65,66]. Whether the amount of Abs generated by elite controllers plays a role in HIV control is currently unknown, but warrants further investigation.

## 5. Conclusions

In summary, we demonstrate here that we can detect and quantify anti-gp120- and anti-HIV Env- specific Abs in untreated progressors, treated progressors and elite controllers. The amount of Ab binding to native trimeric Env is significantly lower than that binding to gp120-coated plates and cCEM. Abs specific to native trimeric Env on HIV-infected cells and open conformation Env on uninfected bystander cells support both ADCC activity. Whether the impact of the Abs to closed conformation HIV Env on HIV control is greater than that of Abs to open conformation Env merits further investigation that will rely on the availability of tools and methods such as those we have described in this report.
